# Alterations in tumor necrosis factor signaling pathways are associated with cytotoxicity and resistance to taxanes: a study in isogenic resistant tumor cells

**DOI:** 10.1186/bcr3083

**Published:** 2012-01-06

**Authors:** Jason A Sprowl, Kerry Reed, Stephen R Armstrong, Carita Lanner, Baoqing Guo, Irina Kalatskaya, Lincoln Stein, Stacey L Hembruff, Adam Tam, Amadeo M Parissenti

**Affiliations:** 1 Regional Cancer Program, Sudbury Regional Hospital, 41 Ramsey Lake Road, Sudbury ON P3E 5J1, Canada; 2 Biomolecular Sciences Program, Laurentian University, L-314, R.D. Parker Building, 935 Ramsey Lake Road, Sudbury, ON, P3E 2C6 Canada; 3 Division of Medical Sciences, Northern Ontario School of Medicine, 935 Ramsey Lake Road, Sudbury, ON P3E 2C6, Canada; 4 Informatics and Bio-computing Platform, Ontario Institute for Cancer Research, 101 College Street, Toronto, ON M5G 1L7, Canada; 5 Faculty of Medicine, Division of Oncology, University of Ottawa, 501 Smyth Road, Ottawa, ON K1H 8L6, Canada

## Abstract

**Introduction:**

The taxanes paclitaxel and docetaxel are widely used in the treatment of breast, ovarian, and other cancers. Although their cytotoxicity has been attributed to cell-cycle arrest through stabilization of microtubules, the mechanisms by which tumor cells die remains unclear. Paclitaxel has been shown to induce soluble tumor necrosis factor alpha (sTNF-α) production in macrophages, but the involvement of TNF production in taxane cytotoxicity or resistance in tumor cells has not been established. Our study aimed to correlate alterations in the TNF pathway with taxane cytotoxicity and the acquisition of taxane resistance.

**Methods:**

MCF-7 cells or isogenic drug-resistant variants (developed by selection for surviving cells in increasing concentrations of paclitaxel or docetaxel) were assessed for sTNF-α production in the absence or presence of taxanes by enzyme-linked immunosorbent assay (ELISA) and for sensitivity to docetaxel or sTNF-α by using a clonogenic assay (in the absence or presence of TNFR1 or TNFR2 neutralizing antibodies). Nuclear factor (NF)-κB activity was also measured with ELISA, whereas gene-expression changes associated with docetaxel resistance in MCF-7 and A2780 cells were determined with microarray analysis and quantitative reverse transcription polymerase chain reaction (RTqPCR).

**Results:**

MCF-7 and A2780 cells increased production of sTNF-α in the presence of taxanes, whereas docetaxel-resistant variants of MCF-7 produced high levels of sTNF-α, although only within a particular drug-concentration threshold (between 3 and 45 n*M*). Increased production of sTNF-α was NF-κB dependent and correlated with decreased sensitivity to sTNF-α, decreased levels of TNFR1, and increased survival through TNFR2 and NF-κB activation. The NF-κB inhibitor SN-50 reestablished sensitivity to docetaxel in docetaxel-resistant MCF-7 cells. Gene-expression analysis of wild-type and docetaxel-resistant MCF-7, MDA-MB-231, and A2780 cells identified changes in the expression of TNF-α-related genes consistent with reduced TNF-induced cytotoxicity and activation of NF-κB survival pathways.

**Conclusions:**

We report for the first time that taxanes can promote dose-dependent sTNF-α production in tumor cells at clinically relevant concentrations, which can contribute to their cytotoxicity. Defects in the TNF cytotoxicity pathway or activation of TNF-dependent NF-κB survival genes may, in contrast, contribute to taxane resistance in tumor cells. These findings may be of strong clinical significance.

## Introduction

Taxanes are a family of chemotherapy drugs used to treat various human cancer types [1-6]. The most common family members include paclitaxel and docetaxel, which block microtubule depolymerization, inducing cell-cycle arrest at mitosis and multinucleation of tumor cells [7,8]. Taxanes also reduce tumor angiogenesis and cell migration, while stimulating the immune system against neoplasms [9-11].

Another mechanism for taxane cytotoxicity may involve tumor-necrosis factor (TNF)-α production, because paclitaxel has been shown to augment TNF-α levels in murine macrophages [12]. TNF-α is a membrane-integrated cytokine (mTNF-α) generally produced in activated macrophages and monocytes [13], which can be released from cells in a soluble form (sTNF-α) by the action of the metalloproteinase ADAM-17 [14]. The release of sTNF-α from cells can then induce cell death or a cell-survival response, depending on the receptor to which it binds: TNFR1 or TNFR2, respectively (reviewed in [15]. Although mTNF-α binds with equal affinity to both receptors, sTNF-α preferentially binds to TNFR1, which has a death-effector domain that induces caspase-8 cleavage and apoptosis [16,17]. Unlike TNFR1, TNFR2 does not contain a death domain. Limited reports suggest that TNFR2 activation promotes cell death, although the mechanism for this is poorly understood and may require the presence of TNFR1 [18,19]. Nevertheless, TNFR2 has been shown to induce NF-κB activity and cell survival [20].

Many mechanisms associated with resistance to taxanes have been identified *in vitro*, such as overexpression of the drug-efflux pump Abcb1, β-tubulin gene mutations, or overexpression of β-tubulin (type III); however, their clinical relevance remains unclear [21,22]. Multiple mechanisms of taxane resistance likely occur simultaneously in cells [23]. To restore tumor sensitivity to taxanes appreciably in cancer patients, all clinically relevant mechanisms of docetaxel resistance must be identified.

To better understand the various pathways associated with taxane resistance, our laboratory selected MCF-7 breast tumor cells for survival in increasing concentrations (doses) of paclitaxel (MCF-7_TAX-1 _cells) [24] or docetaxel (MCF-7_TXT _cells) [23]. Increased Abcb1 expression and decreased taxane uptake occurred on acquisition of taxane resistance in these cells, but a pan-ABC transporter inhibitor that restored taxane uptake had no or only a partial effect on drug sensitivity in these cells [23]. Therefore, additional mechanisms must contribute to taxane resistance, and these cell lines may serve as an attractive tool for assessing the possible role of TNF-α and other pathways in taxane cytotoxicity or resistance.

This study reveals for the first time that docetaxel concentrations of 3 n*M *or greater induce tumor necrosis factor (TNF) expression in MCF-7 cells, and that acquisition of docetaxel resistance can be temporally correlated with elevations in cellular TNF-α levels resistance to TNF-α cytotoxicity, degradation of TNFR1, and promotion of TNFR2-induced survival pathways through the activation of NF-κB. In further support of the role of TNF in taxane cytotoxicity and resistance, we also report that both paclitaxel and docetaxel can induce TNF-α expression in A2780 ovarian carcinoma cells. In addition, we report the consistent alteration in networks of TNF-related genes on acquisition of docetaxel resistance in breast and ovarian tumor cells.

## Materials and methods

### Cell culture and maintenance

MCF-7 cells from the American Tissue Culture Collection (catalog number HTB-22) were cultured or selected for survival in increasing doses of docetaxel or paclitaxel, as previously described [23,24]. The initial concentrations of docetaxel and paclitaxel used to begin selection (dose 1) were 0.51 and 0.56 n*M*, respectively. Cells selected to docetaxel concentrations of 1.11 n*M *(dose 8, MCF-7_TXT8_), 3.33 n*M *(dose 9, MCF-7_TXT9_), 5.00 n*M *(dose 10, MCF-7_TXT10_), 15 n*M *(dose 11, MCF-7_TXT11_), and 45 n*M *(dose 12, MCF-7_TXT12_) were used in this study. Numbers in subscripts of cell-line names refer to the maximal docetaxel dose level to which the cells were exposed. The paclitaxel-resistant cell line used in this study was selected in an identical manner to a final concentration of 6.64 n*M *paclitaxel (MCF-7_TAX-1 _cells; hyphenated number indicates the first cell-line selection, not drug dose). MCF-7 cells were also "selected" in the absence of taxanes to passage numbers similar to those of drug-selected cells to control for genotypic or phenotypic changes associated with long-term culture ("co-cultured control" MCF-7_CC _cells). A2780 ovarian carcinoma cells from the European Collection of Cell Cultures were also selected for resistance to docetaxel in an identical manner (A2780_DXL _cells), including the creation of "co-cultured control" A2780_CC _cells (Armstrong *et al.*, unpublished data).

### Measurement of sTNF-α and sTNFR1 in cell-culture media

Concentrated proteins from the medium of 2 million MCF-7_CC_, MCF-7_TXT_, or A2780 cells (grown in culture in the absence or presence of various concentrations of paclitaxel or docetaxel) were assessed for levels of sTNF-α or sTNFR1 by using ELISA kits from R&D Systems, following the manufacturer's instructions.

### Clonogenic assays

Cellular sensitivity to TNF-α or docetaxel was assessed by using a clonogenic assay, as described previously [24]. Docetaxel resistance factors for the cell lines were determined by dividing the median inhibitory concentration (IC_50_) for docetaxel in the taxane-resistant cell lines by the IC_50 _for MCF-7_CC _cells. In some experiments, cells were exposed to 1 μg/ml cycloheximide, TNFR1 or TNFR2 neutralizing antibodies from R&D Systems (both at 5 μg/ml), or a peptide from Calbiochem Laboratories (La Jolla, CA), which potently blocks NF-κB function by inhibiting translocation of the NF-κB complex into the nucleus [25] (SN-50, 7 μg/ml). A control peptide at the same concentration (SN-50 M) was used in the latter experiments to assess the specificity of NF-κB inhibition.

### Immunoblotting analysis

MCF-7_CC_, MCF-7_TXT_, and MCF-7_TAX-1 _cells were incubated in the absence or presence of 20 ng/ml TNF-α for 24 hours. Cells were extracted in RIPA buffer, and 100 μg of extract proteins assessed for the expression of specific proteins by using standard immunoblotting procedures, as previously described [24]. Antibodies used in these experiments included TNFR1-, TNFR2-, and IκB-specific antibodies from Cell Signaling Technology (Danvers, MA) and a mouse-derived GAPDH antibody from Santa Cruz Laboratories. Densitometric quantitation of bands generated by the IκB antibody was performed by using AlphaEaseFC software (Alpha Innotech, San Leandro, CA). Band intensity was normalized relative to GAPDH band intensity.

### Quantification of TNFR1 and TNF-α transcript levels by RTqPCR

The levels of TNFR1 and TNF-α transcripts in MCF-7_CC _and MCF-7_TXT10 _cells were assessed as described previously [26] by using the following primers: TNFR1: forward, 5'-ACTGCCTCAGCTGCTCCAAAT-3'; reverse, 5'-CCGGTCCACTGTGCAAGAA-3', TNF-α: forward, 5'-TCTTCTCGAACCCCGAGTGA-3', reverse, 5'-GGAGCTGCCCCT-CAGCTT-3'; and S28: forward, 5'-TCCATCATCCGCAATGTAAAAG-3', reverse, 5'-GCTTCTGCGTCTGACTCCAAA-3'.

### Measurement of NF-κB activity

MCF-7_CC _and MCF-7_TXT _cells were cultured in the presence or absence of 50 n*M *docetaxel for 24 hours. The activity of the NF-κB p65 and p50 subunits in 10 μg of nuclear extracts was assessed as outlined in the TransAM NF-κB Family ELISA kit (Active Motif, Carlsbad, CA). Readings at 450 nm were normalized to the sum of all readings on the plate to compare across triplicate experiments.

### Identification of changes in gene expression associated with the acquisition of docetaxel resistance

Agilent 4 × 44 k human genome oligonucleotide arrays were used to profile differences in gene expression between MCF-7_TXT _and MCF-7cc cells at selection dose 10 and between docetaxel-resistant and wild-type A2780 ovarian carcinoma cells (A2780_DXL _and A2780 cells, respectively) at the maximally tolerated dose by using MIAME standards [27]. RNA was isolated from each cell line by using RNeasy Mini kits (Qiagen, Mississauga, ON), and 500 ng of each RNA preparation was labeled and amplified by using Agilent Quick Amp labeling kits. The labeling and array hybridization procedures were performed as per the manufacturer's protocol for a two-color microarray experiment.

### Identification of differences in gene expression associated with docetaxel resistance

The hybridized microarrays were scanned by using Agilent scanners and feature extraction software (version 10_7_3_1), and differentially expressed genes associated with the acquisition of docetaxel resistance were identified by using Partek Genomic Suite (Partek, Inc., St. Louis, MO). The background-corrected intensity values were used for analysis. A three-way ANOVA was performed to identify significant changes in gene expression by using the Method of Moments [28]. Genes with greater than twofold differences in gene expression were selected with a false discovery rate of either 0.05 or 0.01 [29]. The data from these array experiments were deposited in the National Centre for Biotechnology Information Gene Expression Omnibus database (accession number GSE26129) [30].

### Network-based analysis of gene expression

To determine whether the previously described changes in gene expression associated with acquisition of docetaxel resistance in breast or ovarian tumor cells may reflect changes in the function of specific biochemical pathways in these cells, the genes identified as being associated with docetaxel resistance were subjected to functional-interaction (FI) network analysis [31]. In brief, the FI network covers ~50% of the human proteome representing more than 200,000 functional interactions. Pairwise shortest paths among genes of interest in the FI network were calculated and hierarchically clustered (based on the average-linkage method). Clusters were then selected containing more than 90% of altered genes. To calculate a *P *value for the average shortest path, we performed a 1,000-fold permutation test by randomly selecting the same number of genes from the biggest connected network component. A minimum spanning tree algorithm was used to find linkers that connected all genes of interest in one subnetwork [32]. We used the Markov Cluster Algorithm (MCL) [33] with inflation of 1.6 for network clustering. Only the biggest clusters with numbers of proteins not less than 2% of the total network were taken into account. All network diagrams were drawn by using Cytoscape [34]. The functional enrichment analysis for pathways was based on a binominal test. A false discovery rate was calculated based on 1,000 permutations on all genes in the FI network. This network-based analysis was also applied to another dataset that documents differences in gene expression between docetaxel-resistant and parental MDA-MB-231 breast cancer cell lines (Gene Expression Omnibus (GEO) accession number GSE28784).

### Confirmation of microarray-based changes in gene expression by reverse transcription quantitative polymerase chain reaction

A number of the TNF-α-related genes in these networks were further assessed for expression in wild-type and docetaxel-resistant MCF-7 and A2780 cells with reverse transcription quantitative polymerase chain reaction (RTqPCR) by using the primers depicted in Table 1 and the method described earlier.

**Table 1:** Primers selected for confirmation of changes in the expression of tumor necrosis factor-α-related genes by reverse transcription quantitative polymerase chain reaction

Gene	Forward primer	Reverse primer
*S28*	5'TCC ATC ATC CGC AAT GTA AAG-3'	5'-GCT TCT CGC TCT GAC TCC AAA-3'
*TNFAIP3*	5'-GAC CAT GGC ACA ACT CAT CTC A-3'	5'-GTT AGC TTC ATC CAA CTT TGC GGC ATT G-3'
*TNFSF10*	5'-CGT GTA CTT TAC CAA CGA GCT GA-3'	5'-ACG GAG TTG CCA CTT GAC TTG-3'
*TNFSF13*	5'-ACT CTC AGT TGC CCT CTG GTT G-3'	5'-GGA ACT CTG CTC CGG GAG ACT C-3'
*TNFSF14*	5'-TTT GCT CCA CAG TTG GCC TAA TC-3'	5'-CAA TGA CTG TGG CCT CAC CTT C-3'
*TLR1*	5'-GGT ACC AGG CCC TCT TCC TCG TTA G-3'	5'-TAG GAA CGT GGA TGA GAC CG TTT TT-3'
*TLR6*	5'-GCA AAA ACC CTT CAC CTT GTT TTT C-3'	5'-CCA AGT CGT TTC TAT GTG GTT GAG G-3'
*BIRC3*	5'-TGT TGG GAA TCT GGA GAT GA-3'	5'-CGG ATG AAC TCC TGT CCT TT-3'
*TNFR1*	5'-ACT GCC TCA GCT GCT CCA AAT-3'	5'-CCG GTC CAC TGT GCA AGA A-3'
*TNFα*	5'-TCT TCT CGA ACC CCG AGT GA-3'	5'-GGA GCT GCC CCT CAG CTT-3'

Reaction conditions were as described in Materials and methods, and expression was assessed relative to that of *S28*, the internal reference gene.

## Results

### Docetaxel increases sTNF-α production in MCF-7_CC _and A2780 cells

**Figure 1:**
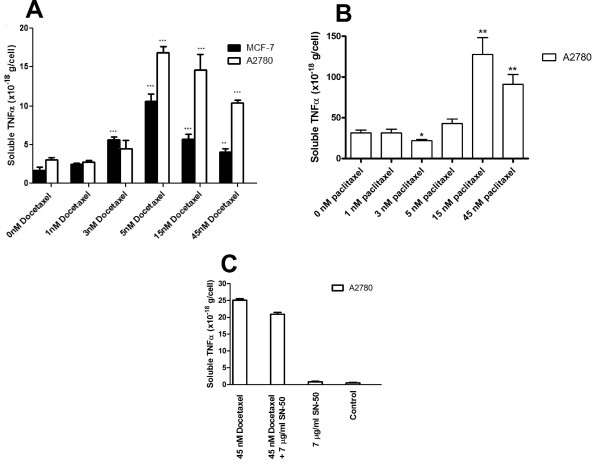
**Docetaxel or paclitaxel-induced production of soluble tumor-necrosis factor alpha (sTNF-α) in MCF-7_CC _and A2780 cells. (a) **The mean concentration of sTNF-α (± standard error) (*n *= 4) found in the medium of MCF-7_CC _(black) or A2780 (white) cells cultured for 48 hours with varying amounts of docetaxel. **(b) **The mean concentration of sTNF-α (± standard error) (*n *= 4) found in the medium of A2780 cells cultured for 48 hours with varying amounts of paclitaxel. **(c) **The effect of docetaxel (45 n*M*) and/or the nuclear factor (NF)-κB inhibitor SN-50 (7 μg/ml) on sTNF-α production in A2780 cells. The significance of differences in sTNF-α levels was assessed by using a Student *t *test; *P *values of < 0.01 and < 0.001 are represented by ** and *** symbols, respectively.

MCF-7_CC _and A2780_CC _cells secreted low levels of sTNF-α (1.69 × 10^-18 ^± 0.40 × 10^-18 ^g/cell and 3.02 × 10^-18 ^± 0.28 × 10^-18 ^g/cell, respectively). These levels were not significantly changed when cells were treated with 0.1 to 1 n*M *docetaxel. In contrast, media extracted from MCF-7_CC _cells treated with ≥ 3 n*M *docetaxel produced significantly elevated levels of sTNF-α (Figure 1a). A2780 cells produced even greater amounts of TNF-α in response to docetaxel (Figure 1a). Interestingly, the taxane paclitaxel (at concentrations ≥ 15 n*M*) induced even higher levels of sTNF-α production than docetaxel in A2780 cells (Figure 1b). Given the stronger induction of TNF-α by docetaxel in A2780 cells, we then assessed whether upstream mechanisms responsible for TNF-α induction in A2780 cells were similar to those of macrophages. Comparable to the induction of TNF-α expression by lipopolysaccharides in macrophages [35], we observed that TNF-α induction by docetaxel in A2780 cells was dependent on NF-κB, because an inhibitor of this transcription factor (SN-50) significantly reduced the induction of TNF-α by docetaxel (Figure 1c). The basal amount of sTNF-α production and the magnitude of docetaxel-induced sTNF-α production varied between experiments (compare Figures 1a and 1c for 45 n*M *docetaxel). Nevertheless, the sTNF-α levels were consistently and substantially higher in cells treated with taxanes. The extent of TNF-α induction by the taxanes appeared to decrease at higher docetaxel concentrations, possibly because of other deleterious effects of these agents on cells at the higher doses.

### Selection of MCF-7 cells in increasing concentrations of docetaxel results in acquisition of progressive docetaxel resistance above a threshold dose

**Figure 2:**
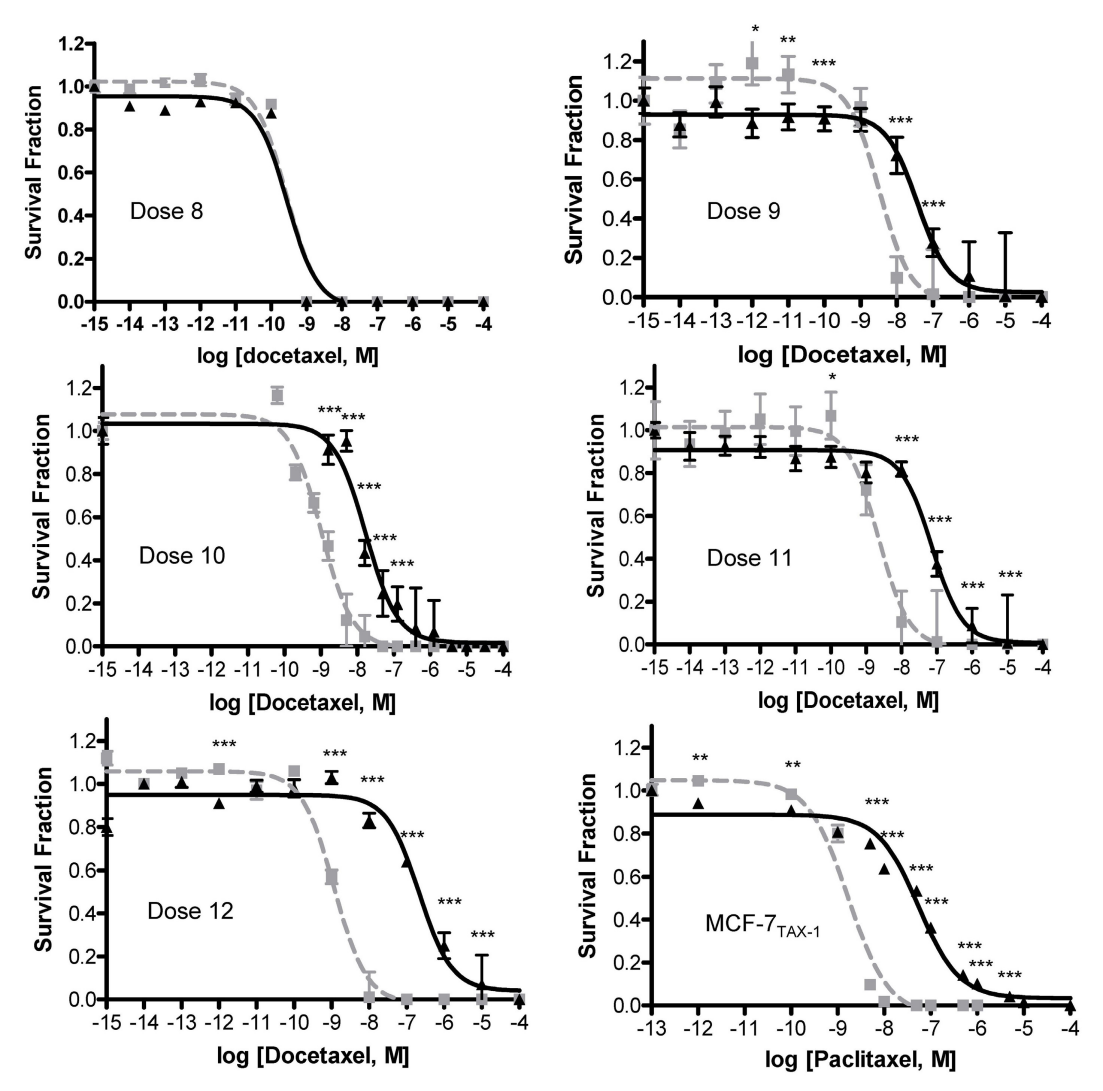
**Acquisition of resistance to docetaxel or paclitaxel in MCF-7 cells**. Sensitivity of MCF-7_CC _cells (broken lines) and taxane-selected MCF-7 cells (solid lines) was measured after selection for survival to dose levels 8 (MCF-7_TXT8_), 9 (MCF-7_TXT9_), 10 (MCF-7_TXT10_), 11 (MCF-7_TXT11_), or 12 (MCF-7_TXT12_), or at the maximally tolerated dose of paclitaxel (MCF-7_TAX-1 _cells). Mean survival fractions (± standard error) are plotted, and the significance of differences in docetaxel sensitivity between the taxane-selected and control cell lines was assessed by using a Student *t *test (*n *= 5); *P *values of < 0.05, < 0.01, and < 0.001 are represented by the *, **, and *** symbols, respectively.

Increasing exposure of MCF-7 cells to docetaxel up to a con{centration of 1.1 n*M *(dose 8, MCF-7_TXT8 _cells) did not affect docetaxel sensitivity (Figure 2). However, selection to 3.33 n*M *docetaxel (dose 9, MCF-7_TXT9 _cells) resulted in an 11.4-fold resistance to docetaxel. Above this threshold, resistance factors increased to 16.6, 32.8, and 184 for cells selected to final docetaxel concentrations of 5 n*M *(dose 10, MCF-7_TXT10 _cells), 15 n*M *(dose 11, MCF-7_TXT11 _cells), and 45 n*M *(dose 12, MCF-7_TXT12 _cells), respectively. Interestingly, MCF-7_TXT _cells exhibited an even greater cross-resistance to paclitaxel, with resistance factors of 148 and 251 at selection doses 11 and 12, respectively [23]. The resistance factor for MCF-7 cells selected for resistance to paclitaxel at the maximally tolerated dose (MCF-7_TAX-1 _cells) was 42. These cells also exhibited strong cross-resistance to docetaxel (46-fold) [24]. In contrast, ovarian A2780 cells could be selected for resistance to considerably higher concentrations of docetaxel. A2780_DXL _cells at their maximally tolerated dose (405 n*M*) exhibited ~4,000-fold resistance to docetaxel (Armstrong *et al.*, unpublished data).

### Effects of docetaxel on sTNFα in MCF-7_CC _and MCF-7_TXT _cell lines

**Figure 3:**
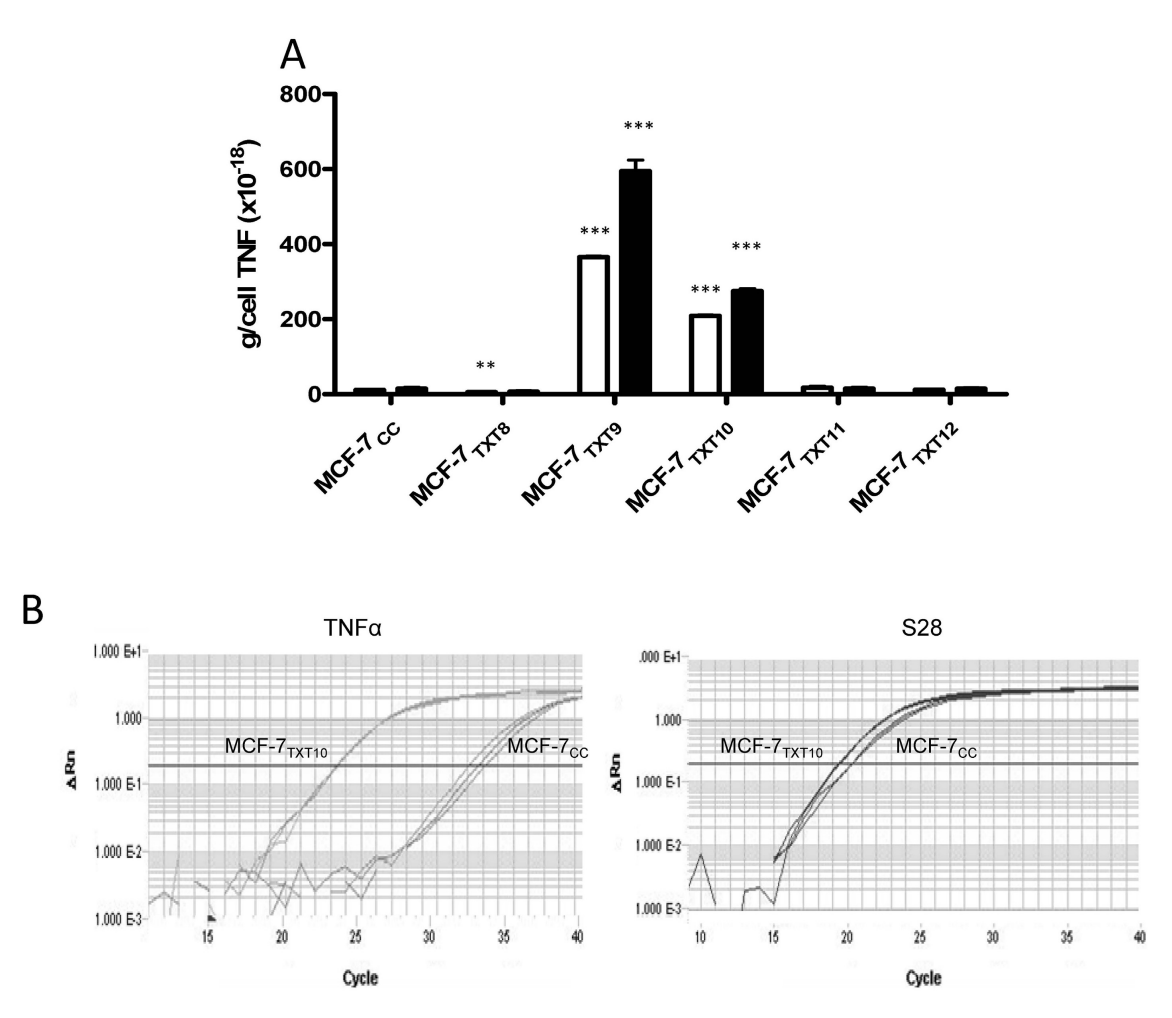
**Levels of soluble tumor-necrosis factor alpha (sTNF-α) in the medium of MCF-7**_CC _**and MCF-7_TXT _cells on exposure to docetaxel**. The ability of MCF-7 cells to produce sTNF-α was measured by using an enzyme-linked immunosorbent assay (ELISA) after selection for survival to docetaxel dose levels 8 (MCF-7_TXT8_), 9 (MCF-7_TXT9_), 10 (MCF-7_TXT10_), 11 (MCF-7_TXT11_), or 12 (MCF-7_TXT12_), or to the maximally tolerated dose of paclitaxel (MCF-7_TAX-1 _cells). After selection, the cells at the various selection doses were assessed for their production of sTNF-α in the absence (white bars) or presence (black bars) of 50 n*M *docetaxel **(a)**. The results presented are the mean levels (± standard error) for five independent experiments, and the significance of differences in sTNF-α levels between MCF-7_CC _and MCF-7_TXT _cells was assessed by using a Student *t *test; *P *values of < 0.01 and < 0.001 are represented by the ** and *** symbols, respectively. **(b) **Expression of TNF-α and S28 transcripts measured with RTqPCR by using cDNA preparations from MCF-7_CC _and MCF-7_TXT _cells (dose level 10).

MCF-7_CC _and MCF-7_TXT8 _cells secreted low amounts of TNF-α (11.5 × 10^-18 ^± 0.4 × 10^-18 ^g/cell and 5.5 × 10^-18 ^± 1.4 × 10^-18 ^g/cell, respectively). When these cell lines were exposed to 50 n*M *docetaxel, no significant difference in sTNF-α secretion was observed (Figure 3a). In contrast, untreated MCF-7_TXT9 _and MCF-7_TXT10 _cells secreted 31.8-fold and 18.2-fold higher levels of sTNF-α than did MCF-7_CC _cells (*P *< 0.0001), and addition of 50 n*M *docetaxel increased sTNF-α production a further 1.62-fold and 1.27-fold, respectively (*P *< 0.01). sTNF-α levels returned to basal levels in MCF-7_TXT11 _and MCF-7_TXT12 _cells, even after treatment with 50 n*M *docetaxel. No differences in sTNF-α levels were observed between MCF-7_CC _and MCF-7_TAX-1 _cells, in the presence or absence of docetaxel (data not shown). TNF-α transcript levels in MCF-7_TXT10 _cells (relative to S28 expression) were 198.5 ± 30.5 higher than the levels of this transcript in MCF-7_CC _cells (Figure 3b), suggesting that elevated secretion of sTNF-α is likely due to dramatically increased expression of TNF-α transcripts and protein.

### MCF-7_TXT _and MCF-7_TAX-1 _cells are resistant to TNF-α-induced cytotoxicity

**Figure 4:**
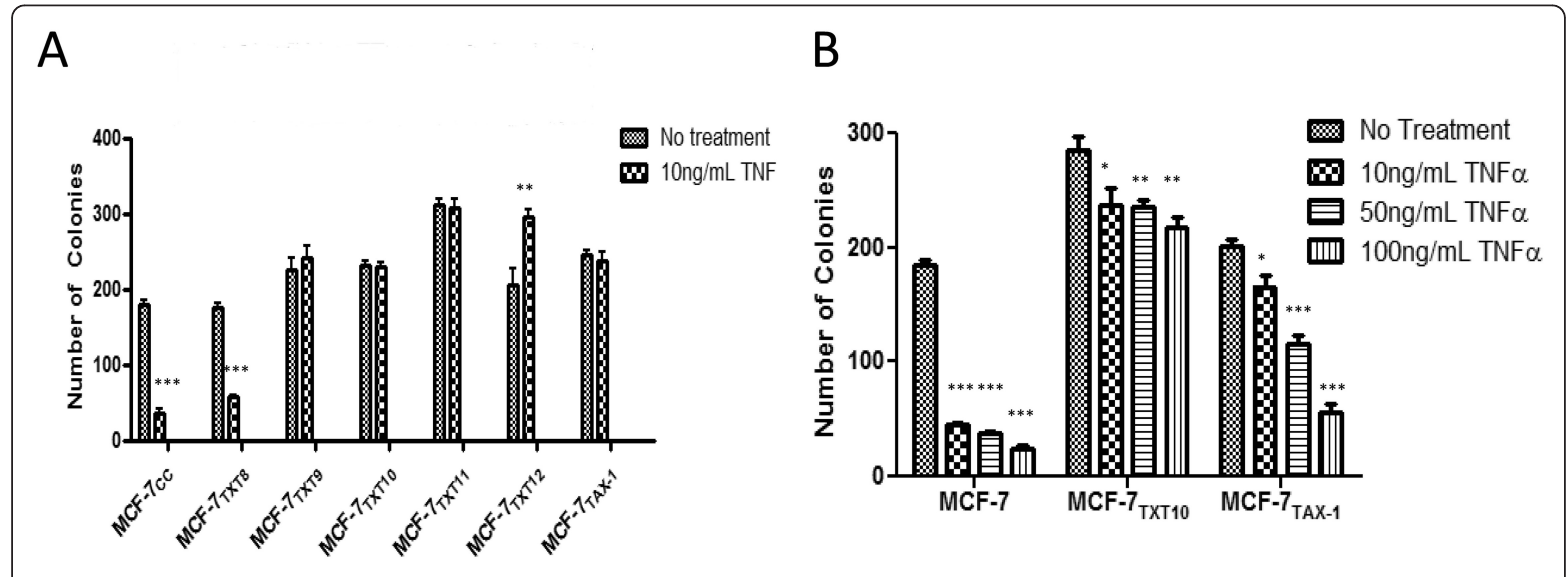
**Sensitivity of MCF-7_CC_, MCF-7_TXT_, and MCF-7_TAX-1 _cells to tumor-necrosis factor (TNF)-α in clonogenic assays**. The effect of docetaxel selection dose on colony formation in MCF-7_CC _and MCF-7_TXT _cells in the absence or presence of 10 ng/ml TNF **(a)**. Colony formation in MCF-7, MCF-7_TXT10_, and MCF-7_TAX-1 _cells in the presence of 0 ng/ml, 10 ng/ml, 50 ng/ml, or 100 ng/ml TNF-α **(b)**. The mean numbers of colonies (± standard error) are depicted, and the significance of differences in TNF-α sensitivity between MCF-7_CC _and MCF-7_TXT _cells at the various selection doses was assessed by using a Student *t *test; *P *values of < 0.05, < 0.01, and < 0.001 are represented by the *, **, and *** symbols, respectively.

TNF-α (10 ng/ml) reduced colony formation in a clonogenic assay by 79.8% ± 6.0% and 66.6% ± 1.7% for MCF-7_CC _and MCF-7_TXT8 _cells, respectively (*P *< 0.0001) (Figure 4a). In contrast, MCF-7_TXT9_, MCF-7_TXT10_, MCF-7_TXT11_, and MCF-7_TAX-1 _cells all had similar levels of colony formation in the absence or presence of 10 ng/ml TNF-α, indicating substantial TNF-α resistance. TNF actually increased colony formation in MCF-7_TXT12 _cells, possibly because of a high level of activation of growth and survival pathways in these cells at the highest selection dose, some of which are TNF-α dependent (see Discussion). The cell lines were also cultured in the presence of varying concentrations of TNF-α. Colony formation was very strongly reduced in MCF-7_CC _cells in the presence of 50 or 100 ng/ml TNF-α (*P *< 0.0001) (Figure 4b). Reductions in colony formation were much smaller for MCF-7_TXT10 _cells treated with 50 ng/ml or 100 ng/ml TNF-α, again indicating resistance to TNF-α cytotoxicity in docetaxel-resistant cells. MCF-7_TAX-1 _cells treated with 10 ng/ml TNF-α formed similar numbers of colonies as did untreated cells, suggesting that these cells were also resistant to TNF-α. However, TNF-α concentrations of 50 or 100 ng/ml induced strong reductions in colony formation relative to MCF-7_TXT10 _cells, suggesting greater resistance to TNF-α in the former cell line than in the latter.

### TNFR1 protein levels (but not transcript levels) are reduced on acquisition of docetaxel resistance in MCF-7 cells

**Figure 5:**
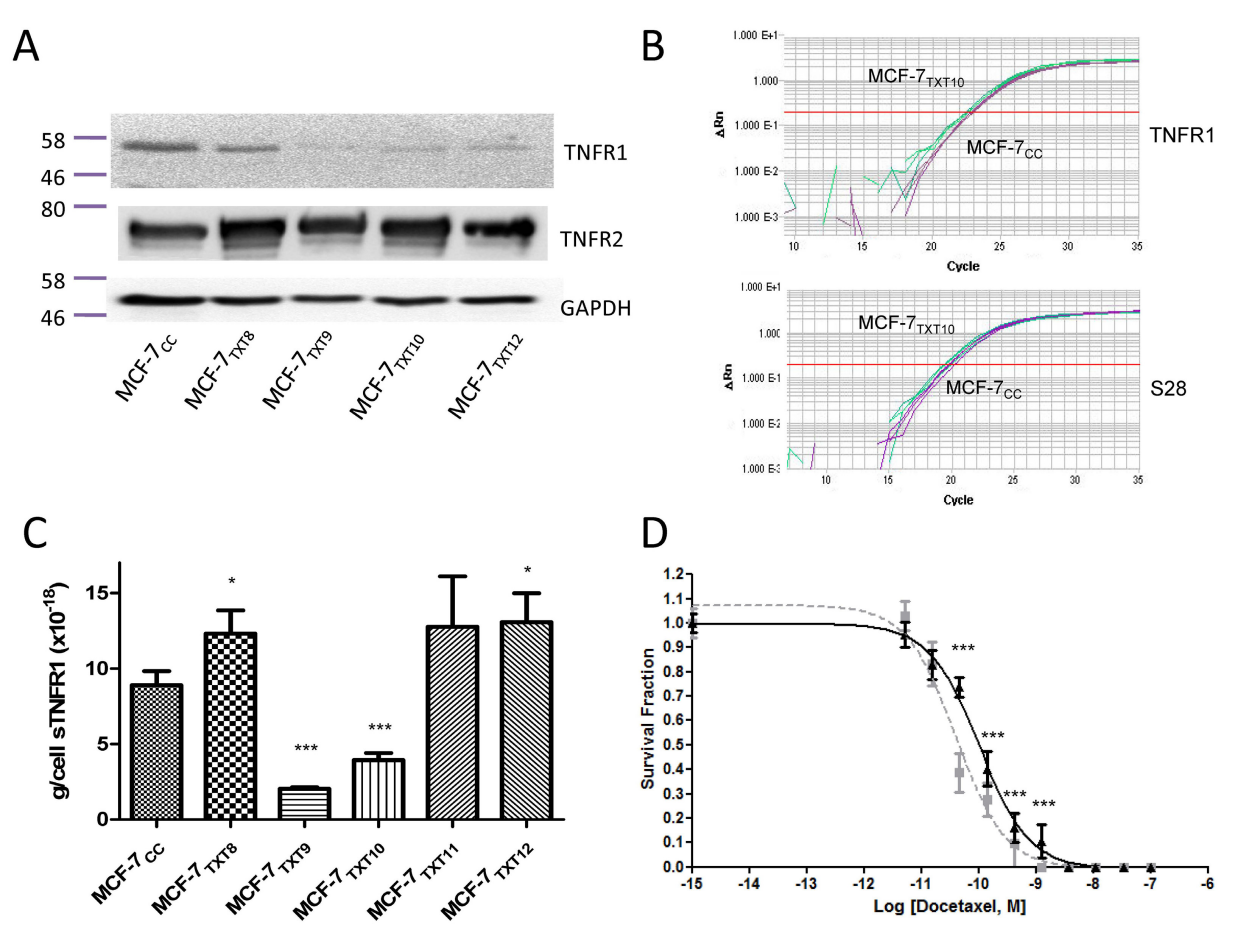
**Tumor-necrosis factor receptor (TNFR)1 and TNFR2 levels in MCF-7_CC _and MCF-7_TXT _cell lines. (a) **Protein levels of TNFR1, TNFR2, and GAPDH in MCF-7_CC_, MCF-7_TXT8_, MCF-7_TXT9_, MCF-7_TXT10_, and MCF-7_TXT12 _cells. **(b) **TNFR1 and S28 transcript levels in MCF-7_CC _and MCF-7_TXT10 _cells determined with reverse transcription quantitative PCR (RTqPCR). **(c) **The mean concentration (± standard error; *n *= 4) of secreted TNFR1 protein was examined in the cell lines by using an ELISA. **(d) **The ability of MCF-7_CC _cells to form colonies in a clonogenic assay at various concentrations of docetaxel in the absence (broken line) or presence (solid line) of 5 μg/ml of a TNFR1 neutralizing antibody (R&D Systems). Error bars represent standard error of the mean. Significance of differences was assessed by using a Student *t *test; *P *values of < 0.05, < 0.01, and < 0.001 are represented by *, **, and *** symbols, respectively.

Unlike TNFR2, the levels of TNFR1 protein (as measured in immunoblotting experiments) decreased on acquisition of docetaxel resistance at dose 9 (MCF-7_TXT9 _cells) and remained low in MCF-7_TXT10 _and MCF-7_TXT12 _cells (Figure 5a). Interestingly, RTqPCR analysis revealed no significant differences in TNFR1 transcript expression between these cell lines (Figure 5b). Similar soluble TNFR1 (sTNFR1) levels were observed in MCF-7_CC _and MCF-7_TXT8 _cells (Figure 5c), although levels decreased in MCF-7_TXT9 _and MCF-7_TXT10 _cells (*P *< 0.001). sTNFR1 levels then returned to those of MCF-7_TXT8 _cells as docetaxel-selective pressure was increased.

### Induction of docetaxel resistance in MCF-7 cells through application of a TNFR1 neutralizing antibody

Significant differences in colony formation were observed between TNFR1 neutralizing antibody-treated MCF-7_CC _cells and untreated cells when incubated with 1.23 n*M *(*P *< 0.0001), 0.41 n*M *(*P *= 0.0002), 0.14 n*M *(*P *= 0.0006), and 0.046 n*M *(*P *< 0.0001) docetaxel (Figure 5d). Nonlinear regression curve-fitting programs revealed that MCF-7_CC _cells incubated with the TNFR1 neutralizing antibody were about 2.25-fold more resistant to docetaxel than were untreated cells, consistent with a role for the TNF-α pathway in docetaxel cytotoxicity.

### Activation of NF-κB on acquisition of docetaxel resistance

**Figure 6:**
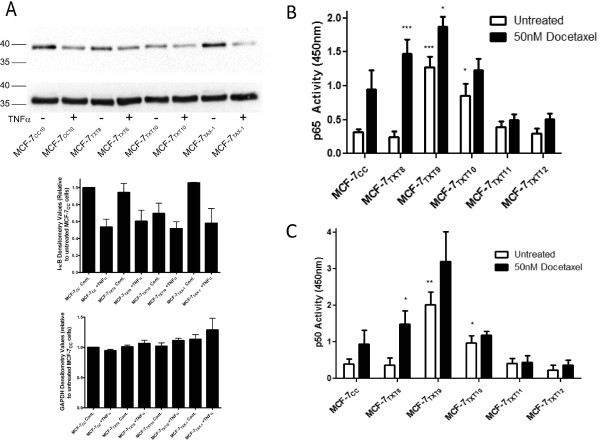
**Nuclear factor (NF)-κB activity in MCF-7_CC_, MCF-7_TXT_, and MCF-7_TAX-1 _cells. (a) **Levels of I-κB and a reference protein glyceraldehyde-3-phosphate dehydrogenase (GAPDH) in MCF-7_CC_, MCF-7_TXT8_, MCF-7_TXT10_, and MCF-7_TAX-1 _cells in the absence or presence of 10 ng/ml Tumor-necrosis factor (TNF)-α. Protein levels were assessed in immunoblotting experiments with quantification of band intensities by using densitometry. The effect of selection for survival in increasing doses of docetaxel on the activity of the NF-κB p65 **(b) **and p50 **(c) **subunits in the absence or presence of docetaxel also was examined by using an ELISA. Mean (± standard error) values are plotted, and the significance of differences between MCF-7_CC _and taxane-resistant cells or differences between treated and untreated cells was assessed by using a Student *t *test; *P *values of < 0.05, < 0.01, and < 0.001 are represented by *, **, and *** symbols, respectively.

Unlike MCF-7_TAX-1 _cells, MCF-7_TXT10 _cells had 35% lower IκB levels than did MCF-7_CC _cells (*P *= 0.03) (Figure 6a). Measurement of NF-κB binding in nuclear extracts from MCF-7 and MCF-7_TXT8 _cells revealed low binding of NF-κB p65 and p50 subunits to the NF-κB transcription factor binding site (Figures 6b and 6c). In contrast, nuclear extracts from MCF-7_TXT9 _and MCF-7_TXT10 _cells exhibited more than threefold higher levels of subunit binding to the NF-κB sequence compared with equivalent extracts from MCF-7_CC _cells (*P *< 0.05). This binding was reduced as cells were exposed to higher docetaxel selection doses. Interestingly, 50 n*M *docetaxel induced even higher levels of p65 and p50 subunit binding in MCF-7_CC _and MCF-7_TXT _cells, except when docetaxel selection doses were more than 15 n*M *(doses 11 and 12).

### Promotion of TNF-α cytotoxicity in MCF-7_TXT10 _cells by cycloheximide or a TNFR2 neutralizing antibody

**Figure 7:**
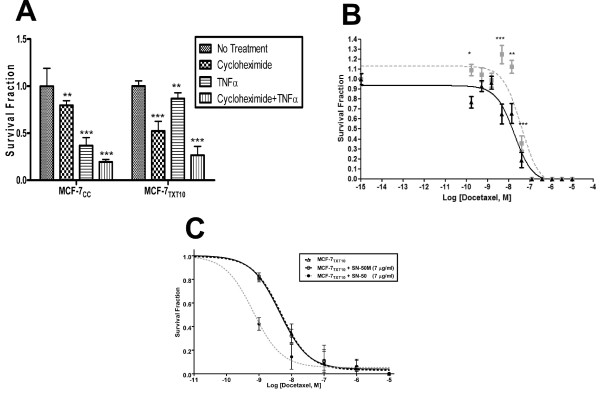
**Effect of various agents on colony formation in MCF-7_CC _or MCF-7_TXT10 _cells**. MCF-7_CC _or MCF-7_TXT10 _cells were assessed for their ability to form colonies after exposure to 10 ng/ml tumor-necrosis factor (TNF)-α, 10 μg/ml cycloheximide, or a combination of both agents for 24 hours **(a)**. The ability of MCF-7_TXT10 _cells to form colonies in increasing concentrations of docetaxel in the absence (broken line) or presence (solid line) of a TNFR2 neutralizing antibody (5 μg/ml) was also examined **(b)**. The effects of an NF-κB inhibitor SN-50 (7 μg/ml; broken gray line) or a control peptide SN-50 M (7 μg/ml; broken black line) on the colony-forming behavior of MCF-7_TXT10 _cells also were examined **(c)**. Mean survival fractions (± standard error) are plotted. Significance of differences was assessed by using a Student *t *test; *P *values of < 0.05, < 0.01, and < 0.001 are represented by *, **, and *** symbols, respectively.

As previously observed, exposure of MCF-7_CC _cells to 10 ng/ml TNF-α strongly decreased colony formation in a clonogenic assay, whereas MCF-7_TXT10 _cells exhibited significant resistance to TNF-α (Figure 7a). The addition of the protein-synthesis inhibitor cycloheximide, 5 μg/ml, restored the ability of TNF-α to be cytotoxic to MCF-7_TXT10 _cells, while having only a small additional effect on TNF-α cytotoxicity in MCF-7_CC _cells. These observations suggested that a protein, possibly NF-κB, is critical for maintaining resistance to TNF-α.

To test this hypothesis, and because NF-κB is activated on TNF-α binding to TNFR2, resulting in enhanced expression of survival genes [20], we theorized that docetaxel cytotoxicity might be increased in MCF-7_TXT10 _cells on addition of a TNFR2-neutralizing antibody or an inhibitor of NF-κB function. Supporting this conjecture, we observed a greater reduction in colony formation for TNFR2-neutralizing antibody-treated cells than untreated cells when treated with 41.2 n*M *(*P *= 0.0007), 13.7 n*M *(*P *= 0.005), 4.5 n*M *(*P *= 0.006), or 1.7 n*M *(*P *= 0.01) docetaxel (Figure 7b). Nonlinear regression curve-fitting for three independent experiments revealed that the TNFR2 neutralizing antibody rendered MCF-7_TXT10 _cells 2.13-fold more sensitive to docetaxel than were untreated cells. Moreover, as shown in Figure 7c the peptide SN-50, which contains the nuclear localization signal of NF-κB and thus blocks the transcription factor translocation to the nucleus [25], increased docetaxel cytotoxicity to an even greater degree in MCF-7_TXT10 _cells (7.1-fold). In contrast, a control peptide (SN-50 M), in which critical basic amino acids within the nuclear localization signal are replaced with uncharged amino acids, had no effect on docetaxel sensitivity (Figure 7c).

### Network-based analysis of genes associated with the acquisition of docetaxel resistance

**Figure 8:**
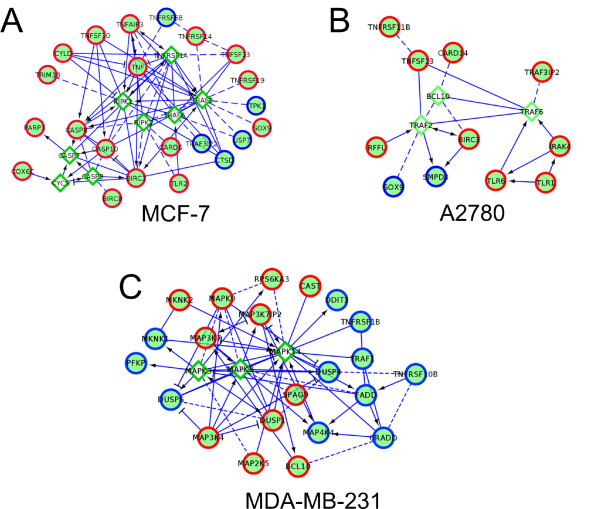
**Networks of tumor-necrosis factor (TNF)-α-related genes that exhibited alterations in gene expression on selection for resistance to docetaxel in MCF-7 breast carcinoma (a), A2780 ovarian carcinoma (b), or MDA-MB-231 breast carcinoma (c) cells**. Gene expressions in the wild-type and docetaxel-resistant cell lines were compared with microarray analysis, after which differentially expressed genes were grouped into functional interaction networks, as described in Materials and methods. Genes upregulated in docetaxel-resistant cells are depicted by using red circles, whereas genes downregulated in docetaxel-resistant cells are depicted by using blue circles. Linker genes are depicted in green diamonds. Direct activating or inhibitory interactions are indicated with the symbols → and ┤, respectively. Indirect interactions involving additional proteins are depicted with dashed lines.

Assessment of microarray data by using an FI network approach (see Materials and methods) revealed 2,235 genes that were differently expressed between parental and docetaxel-resistant MCF-7 breast cancer cell lines (fold-change > 2.0 and FDR ≤ 0.05). Of these, 834 (37.3%) were in the FI network, and hierarchic clustering reduced this to a set of 753 of the most interconnected candidates. This gene set was then used for further analyses. The average shortest-distance calculation showed that genes in this set were linked together much more tightly than would be expected by chance alone (*P *< 0.001), indicating that these differentially expressed genes occupy a small corner of the large FI network space. A subnetwork was built from these 753 genes by adding the minimum number of linker genes required to form fully connected networks involving these genes. The resulting networks consisted of 938 genes, 185 of which were linkers. A Markov clustering algorithm was then used to identify clusters of proteins (coded by the genes) that are highly interconnected with each other and less connected to the outside world. This algorithm identified 14 clusters consisting of more than 20 genes, including a cluster of 22 TNF-associated genes and eight linkers (Figure 8a).

We used an identical approach to identify clusters of differentially expressed genes between wild-type and docetaxel-resistant A2780 ovarian carcinoma cells. Of 955 genes that were differentially expressed between the two cell lines, a network of 11 TNF-related genes and three linkers was identified (Figure 8b). When the same approach was used to identify networks of genes differentially expressed between docetaxel-sensitive and docetaxel-resistant MDA-MB-231 cells (data obtained from GEO, accession number GSE28784), a cluster of 22 TNF-related genes and three linkers was identified (see Figure 8c).

### Confirmation of changes in the expression of TNF-α-dependent genes by RTqPCR

**Figure 9:**
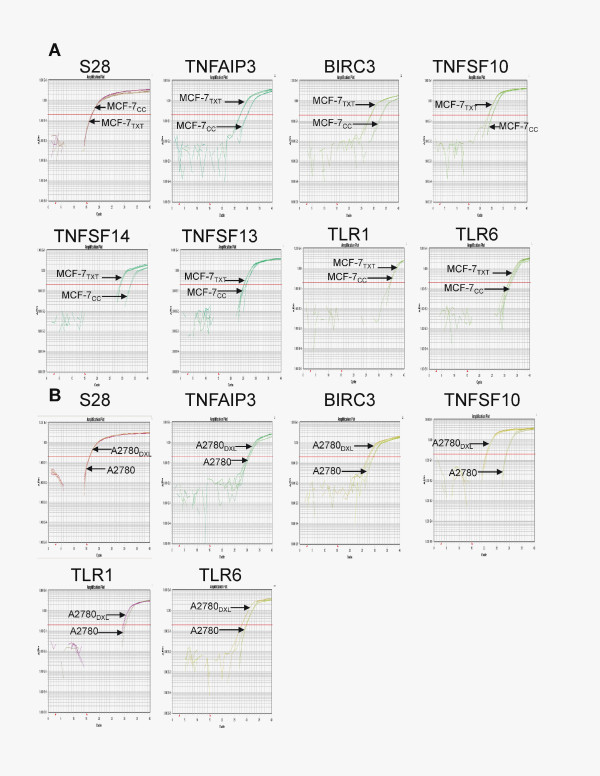
**Use of reverse transcription quantitative PCR (RTqPCR) to assess differences in the expression of Tumor-necrosis factor (TNF-α)-related genes between MCF-7_CC _and MCF-7_TXT _cells (a) and between A2780 and A2780_DXL _cells (b)**. For genes in which qPCR confirmed the changes in gene expression identified by cDNA microarray analysis, representative amplification plots are shown. *S28 *was used as the reference gene.

The expression of a selected number of genes within the previously identified TNF-α signaling networks was quantitatively assessed with RTqPCR. As shown in Figure 9, a generally strong concordance was noted between changes in gene expression identified by microarray analysis and those determined by RTqPCR (12 of 14 gene-expression changes assessed). Six TNF-α-dependent genes were confirmed to have altered expression on selection of MCF-7 cells for resistance to docetaxel, including *TNFSF13, TNFSF10, TLR6, TNFAIP3, TNFSF14*, and *BIRC3 *(the latter two genes being upregulated 30-fold and 21-fold, respectively). Three of these genes were also upregulated in A2780_DXL _cells (*BIRC3, TLR6*, and *TNFSF10*, which increased expression almost 300-fold).

## Discussion

Although taxanes are known to inhibit cell division by preventing microtubule depolymerization and inducing multinucleation [8,36], it is unclear whether these are their sole mechanisms of tumor cell growth arrest/death *in vitro *and *in vivo*. Paclitaxel has been shown to increase sTNF-α release from murine macrophages [12,37], although the levels used in those studies would be unachievable in patients, and docetaxel had no effect on TNF-α expression in the same study. In our study, we showed for the first time that docetaxel (at concentrations between 3 and 45 n*M*) can stimulate TNF-α production and sTNF-α release from both breast and ovarian tumor cells. Such concentrations are clearly in the range of plasma levels of docetaxel in breast cancer patients after docetaxel infusion (10 to 75 n*M*) [38] and are likely sufficiently high to induce TNF expression in even poorly vascularized tumors. This newly identified TNF-dependent mechanism of docetaxel action may also account for its reported immunomodulatory activity [11,39]. In addition, we show in this article that paclitaxel treatment (at 5 and 15 n*M *concentrations) can dramatically increase sTNF-α release from ovarian tumor cells.

Our study also illustrates that the acquisition of docetaxel resistance in breast tumor cells temporally correlates with increased production and release of sTNF-α from cells, despite the ability of sTNF-α to be cytotoxic to cells [40]. However, the onset of docetaxel resistance in MCF-7 cells (at docetaxel selection doses ≥ 3.33 n*M*) also correlated with strongly reduced levels of TNFR1, which would block the ability of TNF-α to induce cell death. Although the mechanism responsible for TNFR1 reduction remains undefined, neither changes in TNFR1 transcript levels nor increased levels of sTNFR1 in the media were found, suggesting that the receptor was not shed from cells by the ADAM-17 protease [14]. In fact, MCF-7_TXT9 _and MCF-7_TXT10 _cells exhibited decreased levels of sTNFR1 in the medium in which it was grown. It is possible that increased levels of sTNFα produced by these cells bound to sTNFR1 in the medium, preventing its detection by the TNFR1 antibody. Taken together, our findings suggest that downregulation of TNFR1 occurs posttranscriptionally, because of either reduced translation of the *TNFR1 *transcript or increased TNFR1 proteolysis.

A recent study [41] found that TNF-α or paclitaxel induced NF-κB activity in C2C12 myotubes. However, paclitaxel did not induce increased TNF-α production, and inhibition of TNFR1 blocked TNF-α-induced NF-κB activation but did not abolish paclitaxel-induced NF-κB activity [41]. It is important to note that, in these studies, TNF-α levels were assessed only 4 hours after treatment with paclitaxel (10 n*M *to 10 μ*M*).

Whereas docetaxel selection doses between 3 and 5 n*M *resulted in highly elevated sTNF-α production, higher selection doses (≥ 15 n*M*) did not. This was despite the ability of the drug to induce TNF-α production in wild-type cells over a large concentration range (Figure 1). This may be explained by the increased expression of the Abcb1 drug transporter and reduced docetaxel uptake that we observed in MCF-7_TXT11 _and MCF-7_TXT12 _cells. Expression was maximal at the highest selection doses (≥ 15 n*M*) [42]. We propose that docetaxel accumulates at sufficient concentrations to induce production of sTNF-α in MCF-7_TXT9 _and MCF-7_TXT10 _cells. However, at or above 15 n*M *docetaxel, MCF-7_TXT _cells exhibit reduced drug uptake, such that docetaxel accumulation is insufficient to stimulate TNF-α production.

The mechanism for resistance to taxanes and TNF-α in MCF-7_TAX-1 _cells appears to differ from that of MCF-7_TXT _cells. TNFR1 levels were equivalent in MCF-7_TAX-1 _and MCF-7_CC _cells (data not shown), and IκB levels were also unchanged during selection for paclitaxel resistance (Figure 6). Because only cells exposed to the maximally tolerated dose of paclitaxel were retained during selection of MCF-7_TAX-1 _cells, it is likely that cells selected at lower doses could have exhibited elevated production of TNF-α and TNF-α-mediated NF-κB activation. However, survival by circumventing the TNF-α ability to stimulate TNFR1-induced cytotoxicity must lie downstream of the receptor. MCF-7_TAX-1 _cells are also high expressors of Abcb1 [24]. Interestingly, another paclitaxel-resistant MCF-7 cell line (MCF-7_TAX-2 _cells) [23] retained sensitivity to TNF-α (data not shown), suggesting that defects in the TNF-α pathway are not critical for taxane resistance *in vitro*. Nevertheless, considering that three of the four taxane-resistant cell lines exhibited alterations in TNF-α signaling and that docetaxel has been shown to increase sTNF-α levels in both breast and ovarian tumor cells, it appears that we have identified a common but unknown mechanism of taxane cytotoxicity and resistance that warrants further study for its potential clinical relevance.

To provide further support for a general involvement of the TNF-α pathway in docetaxel cytotoxicity and in the induction of docetaxel resistance, we also showed in this study that selection of breast and ovarian tumor cells for resistance to docetaxel results in changes in the expression of networks of genes related to TNF-α signaling (Figure 8 and Table 2). Quite strikingly, the vast majority of the upregulated genes depicted in Table 2 code for proteins that are TNF-ligand family members, TNF-receptor family members, TNF receptor-associated proteins, TNF-dependent activators of NF-κB, or proteins that help promote degradation of the inhibitor of NF-κB (IκB). Other upregulated genes are TNF-dependent inhibitors of apoptosis. Downregulated genes code for proteins that inhibit the activation of NF-kB or promote apoptosis. The net effect of the changes in gene expression would thus be to promote the ability of TNF to augment NF-κB-dependent cell survival, while blocking its ability to induce tumor cell death via activation of TNFR1.

**Table 2:** Tumor-necrosis factor α (TNFα)- and nuclear factor (NF)-κB-related genes associated with the acquisition of docetaxel resistance in MCF-7 breast tumor cells, MDA-MB-231 breast tumor cells, and A2780 ovarian carcinoma cells

Gene	Direction and magnitude of change in expression^a ^	Role of gene product	**Refs**.
**Changes in the expression of TNFα- or NF-κB-related genes in MCF-7_TXT10 _cells relative to MCF-7_CC10 _cells**			
*TNF*	Increased 12.6, 1.43	Binds to TNFR1 to promote cell death and to TNFR2 to stimulate expression of NF-κB-dependent survival genes	[54,55]
*TNFSF10*	Increased 6.88	TNF-like ligand that binds to DcR2 to activate NF-κB-dependent survival genes; also called TRAIL	[56]
*TNFRSF19*	Increased 16.5, 1.64	Member of the TNF-receptor family that binds to lymphotoxin alpha, activates NF-κB-mediated transcription	[57]
*TNFRSF14*	Increased, 4.24	TNF-receptor family member, interacts with members of the TNFR-associated factor (TRAF) family and activates NF-κB and AP-1	[58]
*TNFSF13*	Increased, 3.07	Binds to TNFRSF13B to promote NF-κB activation; also called APRIL	[59]
*BIRC3*	Increased, 52.2	Member of a family of proteins that inhibits apoptosis by binding to TNFR-associated factors TRAF1 and TRAF2	[60]
*TLR2*	Increased, 5.19	Stimulates NF-κB activation	[61]
*TRIM38*	Increased, 4.22, 7.28	Removes Lys63-linked ubiquitin chains from TRAF2 and TRAF6, negatively regulating NF-κB activity	[62]
*TNFRSF6B*	Decreased, -2.02	Member of the TNF-receptor superfamily, which, on binding of TNF, inhibits cell proliferation and induces apoptosis	[63]
*TRAF3IP2*	Decreased, -2.14	Associates with and activates I-κB kinase, leading to the liberation of NF-κB from its complex with I-κB	[64]
			
**Changes in the expression of TNFα- or NF-κB-related genes in A2780_DXL _cells relative to A2780 cells (MTD)**			
*TNFSF13*	Increased, 2.10	Binds to TNFRSF13B to promote NF-κB activation; also called APRIL	[59]
*TNFRSF11B*	Increased, 2.01	Decoy receptor for the TNF-related apoptosis-inducing ligand TRAIL; confers resistance to TNF- and TRAIL-induced apoptosis	[65]
*CARD14*	Increased, 2.19, 1.31	Associates with guanylate kinase members that interact with BCL10 and activate NF-κB	[66]
*IRAK4*	Increased, 3.70, 3.86	Required for the optimal transduction of IL-1-induced signals, including the activation of IRAK-1, NF-κB, and JNK	[67]
*TLR1*	Increased, 2.79	Acts via MYD88 and TRAF6 to stimulate NF-κB activation, cytokine secretion, and the inflammatory response	[68]
*TLR6*	Increased, 2.26, 1.57	Also acts via MYD88 and TRAF6 to stimulate NF-κB activation, cytokine secretion, and the inflammatory response	[68]
*BIRC3*	Increased, 8.83	Member of a family of proteins that inhibits apoptosis by binding to TNFR-associated factors TRAF1 and TRAF2	[60]
*RFFL*	Increased, 2.15, 2.13	Endosome-associated ubiquitin ligase for RIP and regulates TNF-induced NF-κB activation	[69]
*TRAF3IP2*	Increased, 2.16	Associates with and activates I-κB kinase, leading to the liberation of NF-κB from its complex with I-κB	[64]
*SMPD3*	Decreased, -1.59, -2.03	Translocates to the plasma membrane in response to TNF-α in a time- and dose-dependent manner	[70]
*SOX9*	Decreased, -30.8	Protein whose expression and activity is negatively regulated by TNF-dependent NF-κB activation	[71]
**Changes in the expression of TNFα- or NF-κB-related genes in docetaxel-resistant MB-231 cells relative to A2780 cells**			
*CAST*	Increased, 2.44	Inhibits degradation of NF-κB to prolong NF-κB activation	[72]
*BCL10*	Increased, 3.48	Potent activator of NF-κB activity	[73]
*RPS6KA3*	Increased, 5.95	Phosphorylates I-κB and activates NF-κB in response to TNF	[74]
*MAP2K5*	Increased, 3.54	A survival protein highly expressed in MCF-7 breast tumor cells resistant to etoposide and TNF-α	[75]
*MKNK2*	Increased, 1.76	Promotes TNF-α biosynthesis at the posttranscriptional levels	[76] [75]
*TNFRSF10B*	Decreased, -1.67	Also known as TRAIL receptor 2; stimulates apoptosis via FADD	[77]
*FADD*	Decreased, -2.00	A component of the caspase 8-activating complex induced by TNF-α binding to TNFR1	[78]
*CRADD*	Decreased, -2.43	An adaptor protein that promotes TNFα-induced apoptosis through interaction with the TNFR1-interacting protein RIP	[79]
*TRAF1*	Decreased, -2.39	Negatively regulates the ability of TNFR2 to promote cell proliferation and NF-κB activation in T cells	[80]
*MKNK1*	Decreased, -2.00	Promotes TNF-α-mediated mRNA degradation	[81]

Full-genome oligo-based microarray experiments were performed comparing differences in gene expression between wild-type and docetaxel-resistant MCF-7 cell lines. Differentially expressed genes were then classified into various functional interaction networks, as described in Materials and methods. Information on the identities and roles of genes associated with TNF-α signaling are presented in tabular form, with particular emphasis on the products of genes that are known to play a role in the induction of NF-κB-dependent survival genes or in the inhibition of apoptosis. **^a^**Numbers represent the fold increase (positive numbers) or decrease (negative numbers) in expression of the gene as revealed by one or more oligo probes on the Agilent 44K human microarrays.

The findings of our study may have significant clinical relevance. A presentation at the 26^th ^annual meeting of the European Association of Urology in 2011 [43] revealed that serum levels of proinflammatory cytokines, including TNF-α, increased 2 days after administration of docetaxel to patients with castration-resistant prostate cancer. Interestingly, these changes in cytokine expression correlated with the induction of apoptosis and with clinical response. In addition, a study presented recently at the American Association for Cancer Research, 101^st ^Annual Meeting [44], revealed that, in patients with serous epithelial ovarian carcinoma, pretreatment tumor expression of various genes within the TNF-α and NF-κB signaling networks could be used to distinguish between responders and nonresponders to paclitaxel/carboplatin chemotherapy. It also was shown in a small study involving patients with locally advanced breast cancer that tumor levels of nuclear (activated) NF-κB could be used to distinguish between responders and nonresponders to neoadjuvant anthracycline- and/or taxane-based chemotherapy regimens [45]. These and other studies strongly support the likely clinical significance of the findings. For example, because TNF-α has been shown to reduce tumor vascularization in mice through its effects on TNFR1-expressing endothelial cells [46], the reported ability of docetaxel to affect tumor angiogenesis [10] may be through an ability of the drug to promote sTNFα-mediated decreases in tumor vascularization. Moreover, one of the well-established dose-limiting toxicities associated with docetaxel chemotherapy in breast cancer patients is fatigue [47], and high TNF levels have been shown to correlate with fatigue onset in cancer patients [48]. Given our findings of docetaxel-induced TNF-α production, perhaps these two phenomena are linked.

Finally, a previous clinical study used a TNF-decoy receptor (entanercept) to permit patients to tolerate higher doses of docetaxel without significant toxicity [49]; however, given our findings, it is not surprising that these blockers would create a greater tolerance to docetaxel, unfortunately at the likely expense of lesser anti-tumor efficacy. Our findings further question the utility of administering docetaxel to cancer patients on TNF-α blockers for treatment of co-morbid inflammatory diseases.

## Conclusions

Our study provides evidence for the first time that taxanes can induce sTNF-α expression in two tumor cell lines of different tissue origin. Although this would promote the cytotoxicity of docetaxel, continued exposure to the drug appears to result in a downregulation of TNF-α-mediated cytotoxicity, while promoting TNF-α-dependent activation of NF-kB-dependent cell-survival pathways and the inhibition of apoptosis. In addition, although drug-resistance studies often involve selection of cells to maximally tolerated drug doses [50,51], our study illustrates the critical role that the drug-selection dose may play on the mechanisms by which tumor cells acquire chemotherapy resistance. At lower doses of taxanes (3 to 5 n*M*), TNF-α-mediated activation of NF-κB-dependent cell-survival pathways appears to be an important mechanism of taxane resistance, whereas at selection doses ≥ 15 n*M *docetaxel, the drug induces overexpression of Abcb1, resulting in reduced accumulation of docetaxel into cells and a consequent reduction in docetaxel-stimulated TNF-α production. Since the concentration of docetaxel within patient tumors is typically lower than that present in the vasculature, perhaps the pathways associated with resistance to lower concentrations of docetaxel are of greater clinical relevance. This may explain why Abcb1 inhibitors have had little ability to reverse resistance to taxanes in cancer patients [52,53]. In addition, given that some cancer patients with inflammatory diseases may be taking TNF blockers, this newly identified role for TNF in docetaxel cytotoxicity may be of particular importance, suggesting that these blockers may compromise the efficacy of docetaxel chemotherapy.

## Abbreviations

A2780_DXL_: docetaxel-resistant A2780 cells (405 n*M*); ANOVA: analysis of variance; DISC: death-inducing signaling complex; ELISA: enzyme-linked immunosorbent assay; MCF-7_CC _cells: co-cultured control MCF-7 cells propagated in the absence of drug; MCF-7_TAX-1_: first MCF-7 cell-line selection with paclitaxel; MCF-7_TAX-2_: second MCF-7 cell-line selection with paclitaxel; MCF-7_TXT8_: docetaxel-resistant MCF-7 cells selected to dose 8 (1.11 n*M*); MCF-7_TXT9_: docetaxel-resistant MCF-7 cells selected to dose 9 (3.33 n*M*); MCF-7_TXT10_: docetaxel-resistant MCF-7 cells selected to dose 10 (5.00 n*M*); MCF-7_TXT11_: docetaxel-resistant MCF-7 cells selected to dose 11 (15 n*M*); MCF-7_TXT12_: docetaxel-resistant MCF-7 cells selected to dose 11 (45 n*M*); mTNF-α: soluble tumor necrosis factor alpha; RTqPCR: reverse transcriptase quantitative polymerase chain reaction; NF-kB: nuclear factor-kappaB; sTNFα: soluble tumor necrosis factor alpha; TNF: tumor necrosis factor; TNF-α: tumor necrosis factor alpha; TNFR1: tumor necrosis factor receptor 1; TNFR2: tumor necrosis factor receptor 2.

## Competing interests

The authors declare that they have no competing interests.

## Authors' contributions

JAS performed the majority of the experiments associated with this investigation and wrote the manuscript. KR performed ELISA and RTqPCR experiments and assisted in preparing the manuscript. SA and CL generated the A2780_DXL _cell line and assisted with microarray experiments. BG conducted the microarray experiments and data analysis and deposited microarray data in the Gene Expression Omnibus database. JAS, SLH, and AT performed the clonogenic assays. IK and LS performed the FI network analyses. AMP participated in the conception and design of the study, assisted in the interpretation of the data, reviewed the manuscript, and made final manuscript revisions. All authors read and approved the final manuscript.

## References

[B1] Sjostrom J Blomqvist C Mouridsen H Pluzanska A Ottosson-Lonn S Bengtsson NO Ostenstad B Mjaaland I Palm-Sjovall M Wist E Valvere V Anderson H Bergh J Docetaxel compared with sequential methotrexate and 5-fluorouracil in patients with advanced breast cancer after anthracycline failure: a randomised phase III study with crossover on progression by the Scandinavian Breast Group Eur J Cancer 1999 35 1194 1201 10.1016/S0959-8049(99)00122-7 10615229

[B2] Shepherd FA Dancey J Ramlau R Mattson K Gralla R O'Rourke M Levitan N Gressot L Vincent M Burkes R Coughlin S Kim Y Berille J Prospective randomized trial of docetaxel versus best supportive care in patients with non-small-cell lung cancer previously treated with platinum-based chemotherapy J Clin Oncol 2000 18 2095 2103 10811675 10.1200/JCO.2000.18.10.2095

[B3] Tannock IF de WR Berry WR Horti J Pluzanska A Chi KN Oudard S Theodore C James ND Turesson I Rosenthal MA Eisenberger MA Docetaxel plus prednisone or mitoxantrone plus prednisone for advanced prostate cancer N Engl J Med 2004 351 1502 1512 10.1056/NEJMoa040720 15470213

[B4] Ajani JA Fodor MB Tjulandin SA Moiseyenko VM Chao Y Cabral FS Majlis A Assadourian S Van CE Phase II multi-institutional randomized trial of docetaxel plus cisplatin with or without fluorouracil in patients with untreated, advanced gastric, or gastroesophageal adenocarcinoma J Clin Oncol 2005 23 5660 5667 10.1200/JCO.2005.17.376 16110025

[B5] Posner MR Docetaxel in squamous cell cancer of the head and neck Anticancer Drugs 2001 12 Suppl 1 S21 S24 11340900

[B6] Iaffaioli RV Tortoriello A Santangelo M Turitto G Libutti M Benassai G Frattolillo A Ciccarelli PD De RP Crovella F Carbone I Barbarisi A Phase I dose escalation study of gemcitabine and paclitaxel plus colony-stimulating factors in previously treated patients with advanced breast and ovarian cancer Clin Oncol (R Coll Radiol) 2000 12 251 255 11005695 10.1053/clon.2000.9167

[B7] Ringel I Horwitz SB Studies with RP 56976 (taxotere): a semisynthetic analogue of taxol J Natl Cancer Inst 1991 83 288 291 10.1093/jnci/83.4.288 1671606

[B8] Chazard M Pellae-Cosset B Garet F Soares JA Lucidi B Lavail Y Lenaz L Taxol (paclitaxel), first molecule of a new class of cytotoxic agents: taxanes Bull Cancer 1994 81 173 181 7894125

[B9] Haldar S Basu A Croce CM Bcl2 is the guardian of microtubule integrity Cancer Res 1997 57 229 233 9000560

[B10] Sweeney CJ Miller KD Sissons SE Nozaki S Heilman DK Shen J Sledge GW Jr The antiangiogenic property of docetaxel is synergistic with a recombinant humanized monoclonal antibody against vascular endothelial growth factor or 2-methoxyestradiol but antagonized by endothelial growth factors Cancer Res 2001 61 3369 3372 11309294

[B11] Tong AW Seamour B Lawson JM Ordonez G Vukelja S Hyman W Richards D Stein L Maples PB Nemunaitis J Cellular immune profile of patients with advanced cancer before and after taxane treatment Am J Clin Oncol 2000 23 463 472 10.1097/00000421-200010000-00007 11039505

[B12] Bogdan C Ding A Taxol, a microtubule-stabilizing antineoplastic agent, induces expression of tumor necrosis factor alpha and interleukin-1 in macrophages J Leukoc Biol 1992 52 119 121 1353517 10.1002/jlb.52.1.119

[B13] Meloni F Tumor necrosis factor alpha. Biological aspects G Ital Chemioter 1989 36 29 37 2488910

[B14] Black RA Rauch CT Kozlosky CJ Peschon JJ Slack JL Wolfson MF Castner BJ Stocking KL Reddy P Srinivasan S Nelson N Boiani N Schooley KA Gerhart M Davis R Fitzner JN Johnson RS Paxton RJ March CJ Cerretti DP A metalloproteinase disintegrin that releases tumour-necrosis factor-alpha from cells Nature 1997 385 729 733 10.1038/385729a0 9034190

[B15] MacEwan DJ TNF ligands and receptors--a matter of life and death Br J Pharmacol 2002 135 855 875 10.1038/sj.bjp.0704549 11861313 PMC1573213

[B16] Grell M Zimmermann G Hulser D Pfizenmaier K Scheurich P TNF receptors TR60 and TR80 can mediate apoptosis via induction of distinct signal pathways J Immunol 1994 153 1963 1972 8051401

[B17] Hsu H Xiong J Goeddel DV The TNF receptor 1-associated protein TRADD signals cell death and NF-kappa B activation Cell 1995 81 495 504 10.1016/0092-8674(95)90070-5 7758105

[B18] Declercq W Denecker G Fiers W Vandenabeele P Cooperation of both TNF receptors in inducing apoptosis: involvement of the TNF receptor-associated factor binding domain of the TNF receptor 75 J Immunol 1998 161 390 399 9647248

[B19] Tartaglia LA Ayres TM Wong GH Goeddel DV A novel domain within the 55 kd TNF receptor signals cell death Cell 1993 74 845 853 10.1016/0092-8674(93)90464-2 8397073

[B20] Rothe M Wong SC Henzel WJ Goeddel DV A novel family of putative signal transducers associated with the cytoplasmic domain of the 75 kDa tumor necrosis factor receptor Cell 1994 78 681 692 10.1016/0092-8674(94)90532-0 8069916

[B21] Monzo M Rosell R Sanchez JJ Lee JS O'Brate A Gonzalez-Larriba JL Alberola V Lorenzo JC Nunez L Ro JY Martin C Paclitaxel resistance in non-small-cell lung cancer associated with beta-tubulin gene mutations J Clin Oncol 1999 17 1786 1793 10561216 10.1200/JCO.1999.17.6.1786

[B22] Mozzetti S Ferlini C Concolino P Filippetti F Raspaglio G Prislei S Gallo D Martinelli E Ranelletti FO Ferrandina G Scambia G Class III beta-tubulin overexpression is a prominent mechanism of paclitaxel resistance in ovarian cancer patients Clin Cancer Res 2005 11 298 305 15671559

[B23] Hembruff SL Laberge ML Villeneuve DJ Guo B Veitch Z Cecchetto M Parissenti AM Role of drug transporters and drug accumulation in the temporal acquisition of drug resistance BMC Cancer 2008 8 318 10.1186/1471-2407-8-318 18980695 PMC2596802

[B24] Guo B Villeneuve DJ Hembruff SL Kirwan AF Blais DE Bonin M Parissenti AM Cross-resistance studies of isogenic drug-resistant breast tumor cell lines support recent clinical evidence suggesting that sensitivity to paclitaxel may be strongly compromised by prior doxorubicin exposure Breast Cancer Res Treat 2004 85 31 51 10.1023/B:BREA.0000021046.29834.12 15039596

[B25] Lin YZ Yao SY Veach RA Torgerson TR Hawiger J Inhibition of nuclear translocation of transcription factor NF-kappa B by a synthetic peptide containing a cell membrane-permeable motif and nuclear localization sequence J Biol Chem 1995 270 14255 14258 10.1074/jbc.270.24.14255 7782278

[B26] Hembruff SL Villeneuve DJ Parissenti AM The optimization of quantitative reverse transcription PCR for verification of cDNA microarray data Anal Biochem 2005 345 237 249 10.1016/j.ab.2005.07.014 16139235

[B27] Brazma A Hingamp P Quackenbush J Sherlock G Spellman P Stoeckert C Aach J Ansorge W Ball CA Causton HC Gaasterland T Glenisson P Holstege FC Kim IF Markowitz V Matese JC Parkinson H Robinson A Sarkans U Schulze-Kremer S Stewart J Taylor R Vilo J Vingron M Minimum information about a microarray experiment (MIAME)-toward standards for microarray data Nat Genet 2001 29 365 371 10.1038/ng1201-365 11726920

[B28] Eisenhart C The assumptions underlying the analysis of variance Biometrics 1947 3 1 21 10.2307/3001534 20240414

[B29] Tamhane AC Dunlop DD Statistics and Data Analysis from Elementary to Intermediate 2000 1 Englewood Cliffs, NJ:Prentice Hall 473 4

[B30] Sprowl JA Armstrong SR Lanner CM Guo B Reed K Hembruff SL Kalatskaya I Stein L Parissenti AM Alterations in tumour necrosis factor signaling pathways associated with cytotoxicity and resistance to taxanes in tumour cells Gene Expression Omnibus Database 2010 National Center For Biotechnology Information http://www.ncbi.nlm.nih.gov/geo/query/acc.cgi?token=hrkztqqskcgsmpu&acc=GSE26129 10.1186/bcr3083 PMC3496117 22225778

[B31] Wu G Feng X Stein L A human functional protein interaction network and its application to cancer data analysis Genome Biol 2010 11 R53 10.1186/gb-2010-11-5-r53 20482850 PMC2898064

[B32] Gross JL Yellen J Graph Theory and Its Applications 1998 1 Boca Raton, FL:, CRC Press

[B33] Enright AJ Van DS Ouzounis CA An efficient algorithm for large-scale detection of protein families Nucleic Acids Res 2002 30 1575 1584 10.1093/nar/30.7.1575 11917018 PMC101833

[B34] Shannon P Markiel A Ozier O Baliga NS Wang JT Ramage D Amin N Schwikowski B Ideker T Cytoscape: a software environment for integrated models of biomolecular interaction networks Genome Res 2003 13 2498 2504 10.1101/gr.1239303 14597658 PMC403769

[B35] Udalova IA Knight JC Vidal V Nedospasov SA Kwiatkowski D Complex NF-kappaB interactions at the distal tumor necrosis factor promoter region in human monocytes J Biol Chem 1998 273 21178 21186 10.1074/jbc.273.33.21178 9694874

[B36] Moos PJ Fitzpatrick FA Taxanes propagate apoptosis via two cell populations with distinctive cytological and molecular traits Cell Growth Differ 1998 9 687 697 9716185

[B37] Manthey CL Qureshi N Stutz PL Vogel SN Lipopolysaccharide antagonists block taxol-induced signaling in murine macrophages J Exp Med 1993 178 695 702 10.1084/jem.178.2.695 8101863 PMC2191120

[B38] Clarke SJ Rivory LP Clinical pharmacokinetics of docetaxel Clin Pharmacokinet 1999 36 99 114 10.2165/00003088-199936020-00002 10092957

[B39] Lee LF Hellendall RP Wang Y Haskill JS Mukaida N Matsushima K Ting JP IL-8 reduced tumorigenicity of human ovarian cancer in vivo due to neutrophil infiltration J Immunol 2000 164 2769 2775 10679119 10.4049/jimmunol.164.5.2769

[B40] Cai Z Capoulade C Moyret-Lalle C mor-Gueret M Feunteun J Larsen AK Paillerets BB Chouaib S Resistance of MCF7 human breast carcinoma cells to TNF-induced cell death is associated with loss of p53 function Oncogene 1997 15 2817 2826 10.1038/sj.onc.1201445 9419972

[B41] Jackman RW Rhoads MG Cornwell E Kandarian SC Microtubule-mediated NF-kappaB activation in the TNF-alpha signaling pathway Exp Cell Res 2009 315 3242 3249 10.1016/j.yexcr.2009.08.020 19732770 PMC3131200

[B42] Reed K Hembruff SL Laberge ML Villeneuve DJ Cote GB Parissenti AM Hypermethylation of the ABCB1 downstream gene promoter accompanies ABCB1 gene amplification and increased expression in docetaxel-resistant MCF-7 breast tumor cells Epigenetics 2008 3 270 280 10.4161/epi.3.5.6868 19001875

[B43] Evans CP Serum levels of pro-inflammatory cytokines immediately increase two days after application of docetaxel in patients with castration-resistant prostate cancer and correlate with apoptosis and clinical response26th Annual Meeting of the Suropean Association of Urology, Vienna, Austria 2011

[B44] Koti M Vidal R Nuin P Haslehurst A Weberpals J Childs T Bryson P Feilloter HE Squire J Park PC Identification of biomarkers of chemoresistance in serous epithelial ovarian cancer using integrative molecular profiling American Association for Cancer Research 102nd Annual Meeting 2011, Abstract 11-A-6026-AACR Orlando, FL

[B45] Montagut C Tusquets I Ferrer B Corominas JM Bellosillo B Campas C Suarez M Fabregat X Campo E Gascon P Serrano S Fernandez PL Rovira A Albanell J Activation of nuclear factor-kappa B is linked to resistance to neoadjuvant chemotherapy in breast cancer patients Endocr Relat Cancer 2006 13 607 616 10.1677/erc.1.01171 16728586

[B46] Stoelcker B Ruhland B Hehlgans T Bluethmann H Luther T Mannel DN Tumor necrosis factor induces tumor necrosis via tumor necrosis factor receptor type 1-expressing endothelial cells of the tumor vasculature Am J Pathol 2000 156 1171 1176 10.1016/S0002-9440(10)64986-3 10751341 PMC1876893

[B47] Schroder CP de ML Westermann AM Smit WM Creemers GJ de GH Stouthard JM van DG Erjavec Z van BA Vader W Willemse PH Weekly docetaxel in metastatic breast cancer patients: No superior benefits compared to three-weekly docetaxel Eur J Cancer 2011 47 1355 62 10.1016/j.ejca.2010.12.018 21251813

[B48] Aouizerat BE Dodd M Lee K West C Paul SM Cooper BA Wara W Swift P Dunn LB Miaskowski C Preliminary evidence of a genetic association between tumor necrosis factor alpha and the severity of sleep disturbance and morning fatigue Biol Res Nurs 2009 11 27 41 10.1177/1099800409333871 19419979

[B49] Monk JP Phillips G Waite R Kuhn J Schaaf LJ Otterson GA Guttridge D Rhoades C Shah M Criswell T Caligiuri MA Villalona-Calero MA Assessment of tumor necrosis factor alpha blockade as an intervention to improve tolerability of dose-intensive chemotherapy in cancer patients J Clin Oncol 2006 24 1852 1859 10.1200/JCO.2005.04.2838 16622259

[B50] Yoo GH Piechocki MP Ensley JF Nguyen T Oliver J Meng H Kewson D Shibuya TY Lonardo F Tainsky MA Docetaxel induced gene expression patterns in head and neck squamous cell carcinoma using cDNA microarray and PowerBlot Clin Cancer Res 2002 8 3910 3921 12473607

[B51] Duan Z Lamendola DE Duan Y Yusuf RZ Seiden MV Description of paclitaxel resistance-associated genes in ovarian and breast cancer cell lines Cancer Chemother Pharmacol 2005 55 277 285 10.1007/s00280-004-0878-y 15565326

[B52] Pusztai L Wagner P Ibrahim N Rivera E Theriault R Booser D Symmans FW Wong F Blumenschein G Fleming DR Rouzier R Boniface G Hortobagyi GN Phase II study of tariquidar, a selective P-glycoprotein inhibitor, in patients with chemotherapy-resistant, advanced breast carcinoma Cancer 2005 104 682 691 10.1002/cncr.21227 15986399

[B53] van der Holt B Lowenberg B Burnett AK Knauf WU Shepherd J Piccaluga PP Ossenkoppele GJ Verhoef GE Ferrant A Crump M Selleslag D Theobald M Fey MF Vellenga E Dugan M Sonneveld P The value of the MDR1 reversal agent PSC-833 in addition to daunorubicin and cytarabine in the treatment of elderly patients with previously untreated acute myeloid leukemia (AML), in relation to MDR1 status at diagnosis Blood 2005 106 2646 2654 10.1182/blood-2005-04-1395 15994288

[B54] Gupta S A decision between life and death during TNF-alpha-induced signaling J Clin Immunol 2002 22 185 194 10.1023/A:1016089607548 12148593

[B55] Karin M Gallagher E TNFR signaling: ubiquitin-conjugated TRAFfic signals control stop-and-go for MAPK signaling complexes Immunol Rev 2009 228 225 240 10.1111/j.1600-065X.2008.00755.x 19290931

[B56] gli-Esposti MA Dougall WC Smolak PJ Waugh JY Smith CA Goodwin RG The novel receptor TRAIL-R4 induces NF-kappaB and protects against TRAIL-mediated apoptosis, yet retains an incomplete death domain Immunity 1997 7 813 820 10.1016/S1074-7613(00)80399-4 9430226

[B57] Hashimoto T Schlessinger D Cui CY Troy binding to lymphotoxin-alpha activates NF kappa B mediated transcription Cell Cycle 2008 7 106 111 10.4161/cc.7.1.5135 18202551

[B58] Marsters SA Ayres TM Skubatch M Gray CL Rothe M Ashkenazi A Herpesvirus entry mediator, a member of the tumor necrosis factor receptor (TNFR) family, interacts with members of the TNFR-associated factor family and activates the transcription factors NF-kappaB and AP-1 J Biol Chem 1997 272 14029 14032 10.1074/jbc.272.22.14029 9162022

[B59] Wu Y Bressette D Carrell JA Kaufman T Feng P Taylor K Gan Y Cho YH Garcia AD Gollatz E Dimke D LaFleur D Migone TS Nardelli B Wei P Ruben SM Ullrich SJ Olsen HS Kanakaraj P Moore PA Baker KP Tumor necrosis factor (TNF) receptor superfamily member TACI is a high affinity receptor for TNF family members APRIL and BLyS J Biol Chem 2000 275 35478 35485 10.1074/jbc.M005224200 10956646

[B60] Liston P Roy N Tamai K Lefebvre C Baird S Cherton-Horvat G Farahani R McLean M Ikeda JE MacKenzie A Korneluk RG Suppression of apoptosis in mammalian cells by NAIP and a related family of IAP genes Nature 1996 379 349 353 10.1038/379349a0 8552191

[B61] Scharf S Hippenstiel S Flieger A Suttorp N N'guessan PD Induction of human {beta}-Defensin-2 in pulmonary epithelial cells by Legionella pneumophila: Involvement of TLR2 and TLR5, p38 MAPK, JNK, NF-{kappa}B and AP-1 Am J Physiol Lung Cell Mol Physiol 2010 298 L687 L695 10.1152/ajplung.00365.2009 20154223

[B62] Brummelkamp TR Nijman SM Dirac AM Bernards R Loss of the cylindromatosis tumour suppressor inhibits apoptosis by activating NF-kappaB Nature 2003 424 797 801 10.1038/nature01811 12917690

[B63] Chen G Rong M Luo D TNFRSF6B neutralization antibody inhibits proliferation and induces apoptosis in hepatocellular carcinoma cell Pathol Res Pract 2010 206 631 641 10.1016/j.prp.2010.05.011 20591579

[B64] Li X Commane M Nie H Hua X Chatterjee-Kishore M Wald D Haag M Stark GR Act1, an NF-kappa B-activating protein Proc Natl Acad Sci USA 2000 97 10489 10493 10962024 10.1073/pnas.160265197 PMC27051

[B65] Rachner TD Benad P Rauner M Goettsch C Singh SK Schoppet M Hofbauer LC Osteoprotegerin production by breast cancer cells is suppressed by dexamethasone and confers resistance against TRAIL-induced apoptosis J Cell Biochem 2009 108 106 116 10.1002/jcb.22232 19544400

[B66] Bertin J Wang L Guo Y Jacobson MD Poyet JL Srinivasula SM Merriam S DiStefano PS Alnemri ES CARD11 and CARD14 are novel caspase recruitment domain (CARD)/membrane-associated guanylate kinase (MAGUK) family members that interact with BCL10 and activate NF-kappa B J Biol Chem 2001 276 11877 11882 10.1074/jbc.M010512200 11278692

[B67] Lye E Mirtsos C Suzuki N Suzuki S Yeh WC The role of interleukin 1 receptor-associated kinase-4 (IRAK-4) kinase activity in IRAK-4-mediated signaling J Biol Chem 2004 279 40653 40658 10.1074/jbc.M402666200 15292196

[B68] Xu Y Tao X Shen B Horng T Medzhitov R Manley JL Tong L Structural basis for signal transduction by the Toll/interleukin-1 receptor domains Nature 2000 408 111 115 10.1038/35040600 11081518

[B69] Liao W Xiao Q Tchikov V Fujita K Yang W Wincovitch S Garfield S Conze D El-Deiry WS Schutze S Srinivasula SM CARP-2 is an endosome-associated ubiquitin ligase for RIP and regulates TNF-induced NF-kappaB activation Curr Biol 2008 18 641 649 10.1016/j.cub.2008.04.017 18450452 PMC2587165

[B70] Clarke CJ Truong TG Hannun YA Role for neutral sphingomyelinase-2 in tumor necrosis factor alpha-stimulated expression of vascular cell adhesion molecule-1 (VCAM) and intercellular adhesion molecule-1 (ICAM) in lung epithelial cells: p38 MAPK is an upstream regulator of nSMase2 J Biol Chem 2007 282 1384 1396 17085432 10.1074/jbc.M609216200

[B71] Rockel JS Kudirka JC Guzi AJ Bernier SM Regulation of Sox9 activity by crosstalk with nuclear factor-kappaB and retinoic acid receptors Arthritis Res Ther 2008 10 R3 10.1186/ar2349 18182117 PMC2374456

[B72] Liu TL Shimada H Ochiai T Shiratori T Lin SE Kitagawa M Harigaya K Maki M Oka M Abe T Takiguchi M Hiwasa T Enhancement of chemosensitivity toward peplomycin by calpastatin-stabilized NF-kappaB p65 in esophageal carcinoma cells: possible involvement of Fas/Fas-L synergism Apoptosis 2006 11 1025 1037 10.1007/s10495-006-6353-y 16547594

[B73] Willis TG Jadayel DM Du MQ Peng H Perry AR bdul-Rauf M Price H Karran L Majekodunmi O Wlodarska I Pan L Crook T Hamoudi R Isaacson PG Dyer MJ Bcl10 is involved in t(1;14)(p22;q32) of MALT B cell lymphoma and mutated in multiple tumor types Cell 1999 96 35 45 10.1016/S0092-8674(00)80957-5 9989495

[B74] Peng C Cho YY Zhu F Xu YM Wen W Ma WY Bode AM Dong Z RSK2 mediates NF-{kappa}B activity through the phosphorylation of IkappaBalpha in the TNF-R1 pathway FASEB J 2010 24 3490 3499 10.1096/fj.09-151290 20385620 PMC2923348

[B75] Weldon CB Scandurro AB Rolfe KW Clayton JL Elliott S Butler NN Melnik LI Alam J McLachlan JA Jaffe BM Beckman BS Burow ME Identification of mitogen-activated protein kinase kinase as a chemoresistant pathway in MCF-7 cells by using gene expression microarray Surgery 2002 132 293 301 10.1067/msy.2002.125389 12219026

[B76] Kotlyarov A Neininger A Schubert C Eckert R Birchmeier C Volk HD Gaestel M MAPKAP kinase 2 is essential for LPS-induced TNF-alpha biosynthesis Nat Cell Biol 1999 1 94 97 10.1038/10061 10559880

[B77] Walczak H gli-Esposti MA Johnson RS Smolak PJ Waugh JY Boiani N Timour MS Gerhart MJ Schooley KA Smith CA Goodwin RG Rauch CT TRAIL-R2: a novel apoptosis-mediating receptor for TRAIL EMBO J 1997 16 5386 5397 10.1093/emboj/16.17.5386 9311998 PMC1170170

[B78] Wang L Du F Wang X TNF-alpha induces two distinct caspase-8 activation pathways Cell 2008 133 693 703 10.1016/j.cell.2008.03.036 18485876

[B79] Ahmad M Srinivasula SM Wang L Talanian RV Litwack G Fernandes-Alnemri T Alnemri ES CRADD, a novel human apoptotic adaptor molecule for caspase-2, and FasL/tumor necrosis factor receptor-interacting protein RIP Cancer Res 1997 57 615 619 9044836

[B80] Tsitsikov EN Laouini D Dunn IF Sannikova TY Davidson L Alt FW Geha RS TRAF1 is a negative regulator of TNF signaling. enhanced TNF signaling in TRAF1-deficient mice Immunity 2001 15 647 657 10.1016/S1074-7613(01)00207-2 11672546

[B81] Rowlett RM Chrestensen CA Nyce M Harp MG Pelo JW Cominelli F Ernst PB Pizarro TT Sturgill TW Worthington MT MNK kinases regulate multiple TLR pathways and innate proinflammatory cytokines in macrophages Am J Physiol Gastrointest Liver Physiol 2008 294 G452 G459 18032482 10.1152/ajpgi.00077.2007

